# P180: The value of real-time sequence based information in surveillance of nosocomial viral infections

**DOI:** 10.1186/2047-2994-2-S1-P180

**Published:** 2013-06-20

**Authors:** J Rahamat-Langendoen, M Lokate, A Friedrich, H Niesters

**Affiliations:** 1 Department of Medical Microbiology, Division of Clinical Virology, University of Groningen, University Medical Center Groningen, Groningen, the Netherlands; 2 Department of Medical Microbiology, Division of Infection Control, University of Groningen, University Medical Center Groningen, Groningen, the Netherlands

## Introduction

Sequence based information is increasingly used in the surveillance of viruses, not only to provide insight in viral evolution, but as a tool to define transmission routes. As most laboratories have not incorporated sequence analysis in their daily routine, information on typing of viruses is mostly available retrospectively. Reducing the time needed to get sequence based information available during an outbreak, should benefit the understanding of transmission routes and guide the implementation of appropriate infection control measures.

## Methods

In August 2012, real time sequencing was introduced at our diagnostic laboratory within the UMCG, a large tertiary referral hospital. A set of viruses, in particular noro-, rhino-, parecho- and enterovirus, is immediately further characterized after detection.

## Results

Sequence analysis results were available within a week after detection. Several clusters of identical subtypes of viruses could be identified, especially in case of norovirus infections. Sequence analysis results confirmed suspected outbreaks based on epidemiological data. However, real-time sequencing also enabled us to rapidly detect a pseudo-outbreak on the children’s oncology ward, in which 5 patients with norovirus infection were notified within one week, despite the implementation of enhanced infection control measures. However, four different genotypes were detected, providing evidence for multiple introductions of different norovirus strains rather than ongoing nosocomial transmissions. This strengthened us to maintain the already implemented infection control measures without closure of the ward.

## Conclusion

Real-time sequence based information, made available immediately after detection, is essential for the understanding of nosocomial transmission of viral infections. This makes it possible to focus infection control interventions.

## Disclosure of interest

None declared

